# Dental Notes in Different Parts of the World

**Published:** 1884-02

**Authors:** George H. Perine

**Affiliations:** New York


### DENTAL NOTES IN DIFFERENT PARTS OF THE WORLD.

BY GEORGE H. PERINE, D. D. S., NEW YORK.

Among those on the continent of Europe who were engaged as practitioners, and investigators, whose object was to establish physiological and pathological fact, we may mention Cuvier and Roussian, who by the aid of the microscope in their researches, contributed much to the increase of professional knowledge. It is to the celebrated naturalist Lieuwenhoek, of Holland, that we are indebted for the discovery of the dental tubuli, which was made by him in 1678.

Ryff, a German, who wrote a valuable work on the teeth, is credited with having, in 1560, invented the "Pelican," an instrument for the extraction of teeth, to which we have frequently referred in previous chapters.

To Purrmann, of Breslau, is given the credit of taking impressions of the mouth in wax as early as 1690, and his was probably the first attempt of the kind made.

Pulpis, a successful practitioner in Amsterdam, a man of much ability, and of experimental mind, recommended the tamponade after the operation of extraction.

The first plaster model of the mouth, it is believed, was made in 1756, by Pfaff, dentist to Frederick the Great.

Nicholas Tulp, an Amsterdam dentist, is said not to have accepted Pare's theory regarding the scarifying of the gums advanced by him in 1569.

Lawrence Heister, a distinguished surgeon and anatomist, who was born at Frankfort in 1683, and practiced in Amsterdam, did not consider lancing of the gums prior to extraction necessary ; he, however, advised the scarifying of the gums in cases of difficult dentition, believing that the convulsions which often accompany this process of nature, are due to the excessive resistance of the gum which is too hard to yield to the pressure of the advancing teeth. His method of removing tumors was to apply to their base a ligature. He also advocated the employment of the actual cautery. For the extraction of different teeth, he recommended various instruments, some of which he manufactured himself. Among those at his command were the "Pelican," "Elevator," and the "Forceps," but of the other appliances we have but meagre descriptions. For filling decayed teeth, Heister employed gold leaf cut into small pieces. In 1707, he was appointed Surgeon-General, and in his connection with the Dutch military hospitals, he performed many important operations during the war in Flanders. Being thoroughly informed in his profession, and a man of much ingenuity, he made many valuable improvements in the instruments then in use. In 1718 he published his celebrated work, "*Institutions of Surgery*," which was in 1739 translated into Latin, and in 1742

into English. It contained forty pages of illustrations, representing various operations, and the instruments employed in their performance He was made Fellow of the Royal Academy of Paris, and in 1719 George I, of England, appointed him Professor of Anatomy and Surgery to the University of Helmstadt, which position he held until his death, which occurred in 1758.

Frank, of Herdelburgh, who was a practitioner of much prominence, and a writer of ability, published in 1672 a work, entitled "*Restoring Teeth to Soundness.*" It was an excellent work for the time in which it was written, and contained many sound theories, which stamped the author as a deep thinker and careful student.

The Germans have for years been making vigorous efforts to establish institutions for the thorough teaching of the dental specialty. Unfortunately, however, the government controls all matters relating to educational interests, hence individual exertions are attended with but slight results. It is, however, believed that movements of importance will be made to induce the government to give its consent and lend its aid to the establishment of such colleges as are so greatly needed, to place at the disposal of those wishing to adopt the specialty, the means of acquiring the necessary knowledge to enable them to practice with credit to themselves and benefit to their patients. Already one of the oldest Universities in Europe—that at Budapest—has established a dental chair, and at Vienna a dental college was founded in 1875, which was the first upon the European continent.

In 1835, a decree was passed by the government of Prussia that "no person should be admitted to examination for a license to practice as a dental surgeon without a good recommendation, and, in addition to his testimonials, he must have attended, during a curriculum of two years, lectures on anatomy ; the theory of medicine ; general, special, and operative surgery ; a chirurgical clinic, when practicable, particularly on dental surgery."

Holland has appointed a dental professorship in one of its medical schools, the Professor of Dental Surgery having passed his dental course with a well known New York practitioner.

In the Netherlands, dentistry was for years restricted by a separate law. In the year 1865, it was decided to make the law uniform, and those wishing to practice dental surgery were compelled to study medicine; the degree of doctor of medicine entitled them to practice the specialty without further study or examination. But few persons, however, directed exclusive attention to the practice of dentistry up to 1878, when, in December of that year, a new law was passed; and, in 1879, additions were made relating thereunto, and regulating the practice of dental surgery, as one of the specialties of medicine.

Germany has its dental organizations; the "Society of German Dentists," being the first, was organized in Berlin in 1859. Since that date nine more have been founded. There are three dental periodicals published in Germany.

What Lieuwenhoek was to Holland, Retzius was to Sweden. He was one of the re-discoverers of the tubular structnre of the teeth. Although Lieuwenhoek, in 1678, and Pur Kingie, in 1735, had announced the tubular character of the human teeth, Retzius, entirely ignorant of the fact, made known after careful investigation and researches (consisting of microscopic examination of human teeth, and those of beasts), that the structure of these organs was of a tubular character. He was one of the great workers in the labyrinth of science, and as such will ever be held in grateful remembrance for the services he has rendered the profession of which he was a valuable member. As an author he had much ability, possessing the faculty of expressing with great clearness his ideas. He was born in 1796. In 1819 he graduated with honors, and was in 1839 appointed professor of anatomy at the Royal Academy, Stockholm. He was also professor of anatomy and physiology at the Royal Caroline Institute. After a brief illness he died on the 18th of April, 1860.

Joel Assur, a dental practitioner in high standing at Stockholm, wrote in 1799, a work on the teeth, which was accepted as a valuable book of reference.

In 1881, a movement, beneficial to the profession, was made in Russia, by the establishment of the dental college of St. Petersburgh.

In Portugal, professional advancement has been by no means as rapid as elsewhere, owing no doubt to the lassitude which pervades every branch of industry in that country, and generally retards progress. Those who desire to practice the profession in Portugal, are obliged to pass an examination before a medical board. Dr. Starbauch, who for a number of years was in practice at Lisbon, endeavored to excite some interest in professional matters with a view to improving the deplorable state of affairs in that city, but with what result we are uninformed. He was in 1856 appointed dentist to the Royal Family.

Spain can boast of one dental publication, and in 1873, a dental department was connected with the Academy of Medicine at Madrid, and in 1880, an American was appointed dentist to the Spanish court.

Italy is credited with a dental organization, known as the "*Soucta Odontologica Italiana.*"

In New Zealand a "Dental Act" similar to that existing in England, went into effect June 1st, 1881. Its requirements are, that those desiring to practice the specialty, shall in addition to the production of credentials establishing their connection with the profession, pass a satisfactory examination before the examining board, which consists of six persons appointed by the University of New Zealand. A heavy penalty is imposed upon those who attempt to practice before having complied with the requirements of this new law.

In Africa, prior to the organization of the American Colonization Society, the practice of dentistry was unknown. We are informed that the most approved method of extracting an aching tooth of any of the natives, was that of attaching to the offending member a string, to the end of which a heavy missile was secured, which upon being thrown with force, generally dislodged the troublesome member, which not unfrequently carried with it a portion of the alveola process. After the establishment of Dr. Clark, at Liberia, and Dr. Tifft, at Kaw Mendi, Western Africa, the popularity of the native method of dental practice began to wane, and the forceps now perform with comparative satisfaction the work of the string and rough projectile.

An act has, however, been passed by Parliament, prohibiting the practice of dentistry, without first having obtained a license from the government, certifying to the proper qualification of those desiring to practice the specialty in Africa.

It is generally conceded that the practice of dentistry in China is of very ancient origin, and the Chinese have been credited with the possession of superior skill in many respects, but we fail to discover upon what ground this theory is based, for the methods adopted by them were in many respects similar to those in favor among the ancient Egyptians, Greeks, and Arabs. Dr. Rogers, however, is of the opinion that the construction and insertion of artificial dentures was understood and practiced in China centuries before the Europeans conceived the idea. The materials employed in manufacturing these substitutes, were bone, ivory, and wood, and the teeth were secured in place with wire. The ancient and modern practice in Japan bears a strong resemblance to that of China.

Rio de Janeiro, the capital of Brazil, has a population of nearly 400,000, and there are, it is stated, nearly two hundred dentists in practice in that city. The Rio de Janeiro Medical College has connected with it a dental department, from which the first graduation was made in 1859.

The University of Bahia, Brazil, also established in 1863 a department of dentistry, from which it will be seen that our South American neighbor is as progressive in his sciences as in industries.

Dr. Dickerson, an American who visited Mexico for the purpose of excavating and making researches among the mounds of its ancient people, informs us that dentistry was undoubtedly extensively practiced by the Aztecs, as fillings and artificial teeth were frequently found by him in the sculls he discovered. At the present day the law requires the practitioner to procure a license from the General Medical Congress, before which body he must undergo an examination which, if satisfactory, entitles him to the privilege of practicing in any part of the republic.

The University of Medicine of the Republic of Mexico, has connected with it a dental department, from which the first American graduated in 1880.

There is one dental journal published in Mexico, and one also in Cuba.

American practitioners can very justly claim the honor of having done much toward raising dental science to a high standard throughout the civilized world. There is scarcely a city of any size upon the face of the globe, where at least one American dentist may not be found, whose beneficial influence is felt and appreciated.

There are in Egypt, Germany, and elsewhere, several female dentists in practice, most of whom are graduates from American schools.

A dental school was established in Switzerland, and located in Geneva in 1880, which is supported by the State.

On the 4th day of July, 1873, a body, consisting of American dentists located in various parts of Europe, was organized in Switzerland, under the title of "The American Dental Society of Europe," and there is little doubt but that this association will continue to exert its influence—which is already great—until throughout the entire continent of Europe the efforts of American skill, energy, and professional enterprise will be still more felt and appreciated.

